# Regulation of growth in *Drosophila melanogaster*: the roles of mitochondrial metabolism

**DOI:** 10.1093/jb/mvaa002

**Published:** 2020-01-11

**Authors:** Howard T Jacobs, Jack George, Esko Kemppainen

**Affiliations:** Faculty of Medicine and Health Technology, FI-33014 Tampere University, Finland

**Keywords:** ecdysone, insulin signalling, PGC-1, proteostasis, pyruvate

## Abstract

**Mitochondrial functions are often considered purely from the standpoint of catabolism, but in growing cells they are mainly dedicated to anabolic processes, and can have a profound impact on the rate of growth. The *Drosophila* larva, which increases in body mass ∼200-fold over the course of ∼3 days at 25°C, provides an excellent model to study the underlying regulatory machinery that connects mitochondrial metabolic capacity to growth. In this review, we will focus on several key aspects of this machinery: nutrient sensing, endocrine control of feeding and nutrient mobilization, metabolic signalling, protein synthesis regulation and pathways of steroid biosynthesis and activity. In all these aspects, mitochondria appear to play a crucial role.**

In the study of animal development, in particular concerning the regulation of growth, the fruit fly *Drosophila* offers a number of advantages compared, *e.g*. with mammals. The use of *Drosophila* is not covered by any ethical restrictions. As it is small and inexpensive to maintain and culture, large numbers of individual organisms can be studied in parallel, allowing for much more statistically robust conclusions, even regarding quantitatively small effects. Having been studied for over a century as one of the classic model organisms, *Drosophila* offers a panoply of genetic and molecular tools enabling fine dissection of gene function and physiology. Moreover, its relatively small genome, with few gene duplications over evolutionary time, avoids the issues arising from gene families and genetic redundancy, which plague studies in most vertebrates. Its development is divided into distinct phases for growth and tissue elaboration, allowing these processes to be studied separately. Importantly, its development gives rise to a very similar set of cell types, tissues and organs as seen in mammals, although many of them are comparatively simpler, allowing developmental processes to be studied in fine detail. Finally, the mitochondrial genetics of *Drosophila* is similar to that of mammals, with maternal inheritance of mitochondrial DNA (mtDNA), encoding an equivalent set of polypeptides as in mammals, and with a very similar apparatus of gene expression.

## Growth in the *Drosophila* Larva

The *Drosophila* larva, a model for the development of holometabolous insects (those undergoing a full metamorphosis) in general, experiences very rapid growth after hatching, at the conclusion of embryogenesis. Within 3 further days at 25°C, the larva reaches a dry weight of ∼0.5 mg, a 200-fold increase over that of the embryo. As in other cases of rapid growth, *e.g*. tumour cells or yeast, the growth of *Drosophila* larvae is primarily fuelled by glycolysis (1). However, this is somewhat of an oversimplification, as discussed in the following section. In regard to growth and nutrition, the life-cycle (after embryogenesis) can be divided into three phases. First, over the three larval instars, biomass is rapidly accumulated, mobilizing food resources for both ATP production and the synthesis of biomolecules (notably proteins and storage lipids: see (2, 3)). At the conclusion of this period, the larva crawls out of the food in which it has been living, and forms a pupa inside a protective case that acts as a barrier to predation, infection and desiccation. In the pupal stage, tissues are extensively reorganized to form the adult organs. As further feeding during this time is not possible, there is a small net decline of biomass, whilst stored lipids are metabolized to provide the energetic needs of cell division, differentiation, migration and eventual eclosion. Finally, the adult is able to feed again: this may be considered a maintenance function in males, whilst in females, the process of oogenesis involves the resumption of significant net biosynthesis to support the development of the next generation.

## Mitochondria, Metabolism and Development

Mitochondria are regarded as the hub of metabolism in almost all eukaryotic cells. Traditionally, the focus has been on the mitochondrial contribution to catabolism, which features centrally in any biochemistry textbook. Three pathways: glycolysis (in the cytosol) and its adjuncts, the beta-oxidation of fatty acids and the breakdown of certain amino acids, converge on the TCA cycle, which is usually portrayed as a machine for completing the oxidation of the key intermediates produced by these pathways. However, the TCA cycle is also essential for biosynthesis. Importantly, because the TCA cycle can be supplied through these diverse primary routes, it acts as a kind of ‘clearing house’, enabling survival and growth on many different primary substrates. Unlike many other metazoans, *Drosophila melanogaster* is considered a cosmopolitan or generalist species, able to survive and thrive on a great variety of different nutritional resources. This is reflected in the confusing diversity of culture media on which the animal is reared in different laboratories. Despite this, the final size and body composition of the adult vary only within a narrow range, except in the case of mutants that are defective in specific metabolic, endocrine or biosynthetic functions, such as ribosome biogenesis or TGF-β signalling (4). The TCA cycle acts as the primary source of the precursors for the synthesis of proteins, lipids, nucleotides and carbohydrates. The biochemical reactions that are responsible for this are typically described as ‘cataplerotic’, meaning that they remove excess materials (from the TCA cycle) that would otherwise accumulate in the mitochondrial matrix. However, these reactions do far more than drain away unwanted metabolites. The extraction of substrates from the TCA cycle before they have been completely metabolized to carbon dioxide and water is a fundamental starting-point for biosynthesis, constituting a physiological role for mitochondria as important as ATP production energized via the respiratory chain (RC). Indeed, in any rapidly growing population of cells, including the *Drosophila* larva, these reactions are the most important function of mitochondria, enabling the processing of carbon skeletons derived from whichever is the predominant food source, for the construction of new biomass. In this process, the oxidative steps of the RC serve mainly an accessory, but still essential function to keep the cycle going. Finally, mitochondrial metabolism serves an additional function, as a major source of heat in all eukaryotes. Biochemical reactions, including those that yield ATP inside mitochondria, are never remotely close to 100% energetic efficiency. Mitochondrial heat production has been studied in specific contexts where it is greatly stimulated, such as in brown fat in mammals or in thermogenic plants, but it has not been explored more broadly in animal development.

The RC and its associated apparatus of oxidative phosphorylation (OXPHOS) are often regarded as the defining feature of mitochondria. This is emphasized by the fact that the most important enzymatic complexes of the RC, as well as the ATP synthase itself, contain subunits encoded in mtDNA, and which are synthesized on mitochondrial ribosomes, constituting an entirely separate translation system in the cell. Moreover, the RNA components of this system are themselves mtDNA-encoded, making the entire synthetic apparatus responsible for sustaining OXPHOS dependent on the molecular machinery of mtDNA maintenance and expression. Any defect in this machinery can therefore impair both bioenergy supply and the provision of raw materials for biosynthesis, as well as influencing mitochondrial heat production, any of which may have detrimental effects on growth. Conversely, as growth processes depend on the TCA cycle which, in turn, is coupled to NADH and ubiquinol oxidation via the RC, the biosynthesis and activity of the RC/OXPHOS complexes should be subject to physiological regulation by the availability of different nutrients, the possible presence of toxic xenobiotics and any other environmental stresses that impact growth. Mitochondrial functions and growth regulation are therefore inextricably linked, especially under conditions where anabolic processes predominate. This short review will consider some documented, extrapolated and hypothesized examples of this linkage, in relation to the growth of the *Drosophila* larva.

## Nutrient Sensing and Preference

A key aspect of larval growth is a ready source of suitable nutrients. The *Drosophila* genus is a good example of how nutrient preference and tolerance evolve, so as to maximize success in different environmental niches. A recent study (5) compared the genetic and biochemical determinants of nutrient tolerance and choice in two *Drosophila* species, *D. sechellia* which lives on one particular nutrient-poor fruit, compared with *D. simulans* which is considered a generalist, with larvae typically found in decaying fruits with a much higher sugar content. These are not mere preferences, as the former species is intolerant to high-sugar diet, whereas the latter does not thrive on low-sugar food sources. Genetic analysis of flies backcrossed under selection revealed two classes of genes responsible for this difference. Genes associated with mitochondrial protein synthesis, such as those coding for mitoribosomal proteins, were down-regulated in sugar-intolerant flies, along with other functions connected with growth and detoxification of xenobiotics (5). Many of these genes were also down-regulated in the sugar-intolerant *mlx^1^* mutant of *D. melanogaster*, which bears a mutation in a key transcription factor responsive to high-sugar diet. RNAi knockdown confirmed the importance, for sugar tolerance, of the genes identified in these studies, and also identified a role for the components of several signalling pathways. The involvement of the mitochondrial translation system mirrors our own findings on *tko^25t^*, a mutant in the gene *technical knockout* (*tko*, encoding mitoribosomal protein S12), discussed in greater detail below, which exhibits slow growth on high-sugar media (6). Both results may seem counterintuitive, in the sense that *a priori* one would predict that flies with down-regulation of mitochondrial respiration would rely more heavily on glycolysis for growth, which would favour high-sugar conditions. A reasonable interpretation is that mitochondrial activity determines the balance between anabolic and catabolic processes. If, under low-nutrient conditions, too great a proportion of the carbon skeletons processed in primary carbohydrate metabolism is routed into the TCA cycle for combustion to CO_2_ and water, there will be insufficient raw materials for biosynthesis, and development may fail. A separate study sheds further light on the way generalist species such as *D. melanogaster* are able to adapt to diets with different carbohydrate contents, via TGF-β signalling (7). In this case, the TGF-β family member Dawdle acts on the gut and other tissues to repress the expression of many genes involved in carbohydrate metabolism, decreasing the toxic effects of sugar overload (7).

## Neural and Endocrine Control of Feeding and Nutrient Usage

Perhaps surprisingly, genetic and metabolic influences on feeding behaviour have been studied more extensively in *Drosophila* adults than larvae. Foraging behaviour is under complex genetic, neural and even social control, enabling larvae to respond to cues such as odour, sweetness and stiffness of the food substrate (8). Nutrient intake is regulated by a complex network of neuropeptides, some of them interacting with the insulin-signalling pathway described below (9). Some also regulate other behaviours, and are expressed in (and stimulate) very restricted sets of neurons (10). Although a specific mitochondrial interaction with this circuitry has not been documented, mitochondrial metabolism clearly plays a role in neurotransmission via the TCA cycle intermediate α-ketoglutarate, a sufficient level of which is needed for fusion of synaptic vesicles (11). Another link between metabolism and feeding intensity involves the secretion of tyramine as a negative regulator of motoneuron excitability, following feeding (12). Upon starvation, tyramine levels drop, resulting in increased locomotion. Although mitochondria are not involved in the synthesis of biogenic amines, the mitochondrially located monoamine oxidases are responsible for their oxidative deamination, releasing hydrogen peroxide. Thus, mitochondrial metabolism is involved in the signalling that underlies this crucial behavioural switch.

*Drosophila* has a binary hormonal system considered equivalent to glucagon and insulin in vertebrates. Adipokinetic hormone functions like glucagon (though the two peptides are not structurally similar), promoting the breakdown of carbohydrate stores and raising the haemolymph sugar level. The insulin/insulin-like growth factor signalling (IIS) system is both simpler than in vertebrates, with a single receptor and response pathway (InR, PI3K, Akt, FOXO) encoded by single-copy genes, but is also more complex, with an array of 8 insulin-like peptides (DILPs) and a number of (mostly antagonistic) regulatory peptides deployed in a stage- and tissue-specific manner (see (9, 13) for review). In the larva, DILPs regulate growth and nutrient usage in the tissues, both by modulating the transcriptional activity of FOXO, and via the mTORC1 complex that controls the rate of protein synthesis and is subject to various additional metabolic inputs (discussed in a later section).

A number of studies have profiled effects of high-sugar diet on wild-type flies, with chronic hyperglycaemia, peripheral insulin resistance, developmental delay and increased amounts of stored lipids as the main phenotypic outcomes (14). A crucial organ in the fly that mediates responses to carbohydrate overload is the fat body (insect equivalent of the vertebrate liver and adipose tissue), in which the transcription factor Seven-up has been implicated as a key component of the regulatory machinery that integrates the antagonistic effects of insulin and steroid signalling (15). Seven-up also links glucose metabolism with antimicrobial immunity, in which a recently characterized long non-coding RNA has been implicated (16). In addition, the fat body responds to amino acid starvation by emitting an (as yet unidentified) endocrine signal that down-regulates growth in the entire larva (17). Some neuropeptides synthesized in the fat body create a feedback loop to the insulin-producing cells of the brain, down-regulating insulin signalling in response to nutrient deprivation (18).

The role played by mitochondria in these various pathways remains unclear. RC dysfunction or overload increases the production of reactive oxygen species (ROS) which, in turn triggers various anti-growth responses. Amongst them are the activation of a checkpoint mechanism that blocks cell-cycle progression from G1 to S phase, converging with and thus counteracting IIS at the level of FOXO (19), as well as via a growth inhibiting signal acting on the global translational regulators 4EBP and S6 kinase (S6K) (20).

## Metabolic Signalling

On a basic level, numerous studies have shown that mitochondrial metabolism, including the genetic apparatus that underpins it, is essential for larval growth. Mutants with decreased capacity for OXPHOS show three characteristic phenotypes: decreased larval growth rate, manifesting as delayed eclosion, bang-sensitivity (seizures induced by mechanical stress) and impaired responsiveness to sound. In addition to *tko^25t^*, such phenotypes have been reported for *sesB^1^*, which bears a non-silent mutation in *stress-sensitive B*, encoding the major adult isoform of the adenine nucleotide translocase, and *kdn^PC64^* carrying a point mutation in *knockdown*, coding for the major isoform of citrate synthase. The *tko^25t^* mutant phenotype also includes impaired male courtship. Although the mechanistic connections between mitochondrial dysfunction, bang-sensitivity, deafness and defective male courtship remain unclear, these phenotypes can all be corrected by transgenic expression of the wild-type *tko* gene (21), and are therefore attributable to the specific point mutation in *tko^25t^*. Note, however, that effects of genetic background cannot be excluded. In fact, they are strongly suggested by the fact that the severity of the *tko^25t^* phenotype is alleviated by long-term inbreeding (21), as well as by duplication of the mutant gene in its natural chromosomal milieu (22), though is unaffected by an ectopically inserted copy of the mutant allele (21). Furthermore, mtDNA background can dramatically modify the phenotype, ranging from partial suppression (23) to lethality (24).

Some progress has been made in understanding how limitations on the metabolic capacity of mitochondria influence growth rate in fly larvae. One key finding is that the developmental delay of *tko^25t^* can be partially alleviated by expressing wild-type *tko* in any of a variety of different tissues, using the UAS/GAL4 system, whilst a partial developmental delay can conversely be caused by RNAi knockdown of *tko* in any of several specific tissues (25). As there is little or no overlap between the patterns of expression of the various drivers used in these experiments, it follows that mitochondrial dysfunction is somehow monitored or integrated across the entire larva, behaving in some ways as a single cell at the metabolic level. In other words, a limited capacity for mitochondrial metabolism in neurons is somehow compensated by mitochondrial metabolism in muscle, fat body or gut being normal and so on. This result makes sense in that the growth of all tissues needs to be coordinated, to produce the fully formed and properly scaled larva that is competent to undergo metamorphosis. It implies that either one or more crucial metabolites are shared systemically and/or that endocrine signalling is integrated across the different tissues of the larva to modulate growth rate according to resources and the capacity of mitochondria to use them.

If, instead, each tissue were metabolically autonomous, then an external insult, such as a toxin absorbed from the environment, which affects metabolism in only one or a small subset of tissues, could potentially lead either to a catastrophic loss of developmental synchrony or to a drastic, global deceleration of development. Whether the integrative system is metabolic, endocrine or both, the rates of protein synthesis and secretion, which are the key processes underlying growth, must be attuned in different tissues to the abundance of the raw materials for biosynthesis: energy (in the form of ATP and GTP), reducing equivalents (in the form of NADPH), and amino acids. Much evidence indicates that all three control the rates of protein synthesis through a network of protein kinases acting directly on the translational machinery at several levels, as discussed in a following section. First, we will consider the nature of metabolites that may be shared across the larva or contribute to growth-regulatory signalling. 

**Fig. 1.**
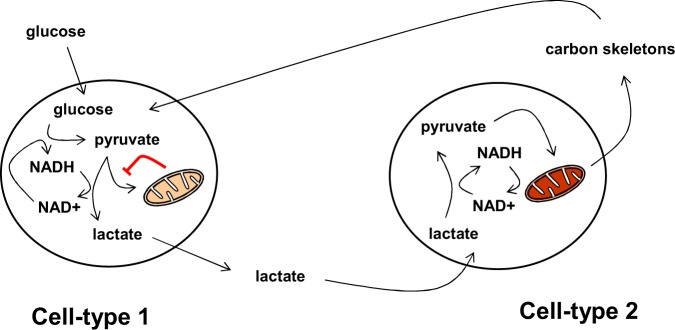
**Hypothetical scheme for metabolite sharing to alleviate the burden of mitochondrial dysfunction or overload in a given cell type.** In cell-type 1, glucose is metabolized by glycolysis, but the capacity of mitochondria to process the resulting pyruvate (and NADH) is limited (denoted by the pale brown colour). Instead the pyruvate is mostly converted to lactate, in the process also regenerating NAD+. Lactate excreted from cell-type 1 is imported into cell-type 2, where mitochondrial capacity is higher (denoted by the darker brown colour), allowing it to be fully processed, yielding carbon skeletons for biosynthesis, also in cell-type 1. NADH is indirectly imported into mitochondria in cell-type 2 via the shuttle system, and converted back to NAD+ by the RC. A similar type of metabolic co-operation is hypothesized in tumours, whereby lactate is secreted from cells at the core of the tumour, due to the inhibition of mitochondrial functions by hypoxia, but is then processed by less hypoxic cells at the tumour periphery.

A shared metabolite should be relatively abundant, occupy a central place in metabolism, and be sufficiently non-reactive to be stable when secreted into the extracellular space and transported to distant tissues. It should also be found at abnormal levels in larvae experiencing a growth defect due to metabolic insufficiency, such as in the in *tko^25t^* mutant. Based on metabolite analysis of third-instar (L3) *tko^25t^* larvae (6), we can consider various hypotheses. Apart from specific amino acid deficiencies, which seem to be ruled out as instrumental by virtue of the fact that dietary yeast supplementation had no effect on the developmental delay of *tko^25t^*, there were no major metabolites at abnormally low levels in mutant larvae, other than the relatively unstable ‘high-energy’ nucleotides ATP and NADPH. However, pyruvate and lactate, at the end-point of glycolysis, are at abnormally high levels in *tko^25t^* larvae, raising the possibility that either could be a surrogate for the insufficiency of mitochondria to process carbon-skeletons. Lactate also accumulates to high levels in the haemolymph of *tko^25t^* larvae (6), making it a strong candidate for being the intermediate that shares the burden of mitochondrial insufficiency throughout the larva, as illustrated in Fig. 1. This concept was strengthened by the observation that high concentrations of dietary pyruvate or lactate exacerbate the developmental delay of *tko^25t^*, and even phenocopy the mutation to a considerable extent in wild-type flies (6, 25). However, drugs that alter pyruvate levels in opposite ways both exacerbated the phenotype of *tko^25t^* (25), whilst knockdown of genes encoding various enzymes of pyruvate metabolism produced significant, but partially contradictory effects on the development of wild-type and mutant larvae. Knockdown of lactate dehydrogenase, the key enzyme involved in lactate–pyruvate interconversion, was lethal to both wild-type and *tko^25t^* larvae (25). On balance, these findings strongly suggest that a metabolic derivative of pyruvate, though not, at least not solely, pyruvate itself, is involved in metabolite sharing and or signalling in response to mitochondrial capacity. However, the exact mechanism remains to be elucidated. In principle, abnormal levels of pyruvate and/or its metabolites may restrain growth by acting directly on metabolic transactions, by signalling to the machinery of protein synthesis as discussed in the following section, or by influencing epigenetic processes in the nucleus. In this regard, lactylation of histones has recently been reported in mammalian cells, where it can influence gene expression and cellular physiology (26).

## Protein Synthesis Regulation

The link between mitochondrial function and cytosolic protein synthesis in the *tko^25t^* mutant has also not been fully worked out, but the basic mechanisms regulating protein synthesis in response to metabolic signals are common to all eukaryotes and have been extensively characterized in *Drosophila*. Amino acid availability is monitored through the mTOR (target of rapamycin) complex, whilst the primary sensor of cellular energy charge is AMPK. The identity of the NADPH-dependent regulator is less certain, but circumstantial evidence strongly implicates Sik3 (salt-induced kinase 3).

*tko^25t^* larvae exhibit abnormal levels of the known or putative activators of all three pathways: low ATP, low NADPH and both elevation and depletion of specific amino acids, *i.e*. high alanine and serine, but low aspartate, asparagine and histidine (6). Progress in elucidating how these metabolites and their cognate regulators are involved in growth regulation in *tko^25t^* is hampered by the fact that the key catalytic subunits of almost all of the relevant, known regulators are encoded by essential genes.

The mTOR kinase, acting together with its key modifiers Raptor (in the mTORC1 complex) and Rictor (in the mTORC2 complex) is a conserved regulator of growth and cell proliferation in response to nutritional and metabolic signals and other stimuli. mTORC1 promotes growth by activating ribosomal protein S6K and inhibiting the translational repressor 4EBP, thereby increasing the rate of cytosolic protein synthesis (see (27) for review). A more thorough discussion of the mTOR complexes is beyond the scope of this article, but a handful of examples will suffice to show how mTOR signalling is linked to the other pathways described here. The adaptor proteins Unkempt and Headcase, which interact with the Raptor subunit of mTORC1, mediate responses to nutrient deprivation by regulating S6 phosphorylation in an insulin-dependent manner (28). Regulation of S6K at the protein level, which we documented in *tko^25t^* larvae grown on high-sugar medium (6), has recently been shown to depend on a specific E3-ubiquitin-ligase, Archipelago (29), which may represent a key part of the bridge between metabolic signalling and protein synthesis regulation.

AMPK in *Drosophila*, as elsewhere, serves primarily as an energy sensor to adjust the relative rates of ATP production and utilization (see (30) for review). Unlike many other organisms, the three subunits of the AMPK heterotrimer are encoded in *Drosophila* by single-copy genes, enabling a clearer genetic analysis of AMPK function in development and physiology. AMPK signalling is a crucial regulator of larval feeding and foraging behaviour in response to diet (31), as well as an upstream modulator of mTORC1 signalling, via the inhibitory effect produced by phosphorylation of Raptor and Gigas (Tsc2) (32). The AMPK–TSC2–mTORC1 pathway is required for growth regulation during metamorphosis (33), and has been shown to control cell division in imaginal discs at L3, in response to ATP deficiency caused by a mitochondrial OXPHOS defect (34).

AMPK also regulates several processes in oogenesis in response to dietary stress (35), notably restraining follicle cell proliferation which then acts indirectly on the germline. AMPK has an additional, basal signalling function in germline stem cells under normal nutritional conditions (35).

Sik3 is a homologue of AMPKα, although it is not known if it associates with other polypeptides structurally or functionally homologous with the β and γ subunits of AMPK. In developing flies it maintains the ratio of NADPH and NADP+ in response to dietary sugar, by stimulating the activity of glucose-6-phosphate dehydrogenase (Zwischenferment), the rate-limiting enzyme of the pentose phosphate shunt (36). NADPH is essential for the reduction of glutathione. Sik3 therefore provides resistance against oxidative stress under high-sugar conditions (36). Other studies have implicated it as a driver of cell growth and tumourigenesis via down-regulation of the Hippo signalling pathway on high-sugar diet (37, 38). Sik3 interacts directly with Salvador, one of the scaffold proteins for the Hippo kinase complex (37). This provides a potential link between Sik3 and S6K regulation, as Hippo and S6K have both identified as interaction partners for Archipelago (39, 40). Sik3/Hippo also regulates the growth programme at the transcriptional level, via the transcriptional co-activator Yorkie (37), and this has been shown to respond to the functional state of mitochondria (41).

An additional kinase, LKB1, acts upstream of both AMPK and Sik3. Some functions of LKB1, *e.g*. in mitosis and cell polarity, involve only AMPK (30), whereas its lipid storage-promoting functions are mediated through Sik3 (42) and modulated by diet via the insulin signalling pathway. Sik3 is also regulated via the sugar-sensing Mondo–Mlx complex (36). Because of its structural similarity to AMPKα and its action to regulate at least one key enzyme involved in NADPH production, it is tempting to suggest that it could be regulated in a similar manner as AMPK, *e.g*. via the ratio of NADPH to NADP. The adenine nucleotide-binding subunit of AMPK (AMPKγ) contains several binding sites for nucleotides, one of them also capable of competitively binding NADPH (43). In *Drosophila* as in other species, AMPKγ (*SNF4Aγ*) is expressed as a number of splice-variants, therefore some of the encoded proteins may be considered candidates for direct (or indirect) interaction with Sik3, although this remains to be tested.

A clear indicator of regulation in *tko^25t^* larvae at the level of cytosolic translation comes from inhibitor studies. Whilst *tko^25t^* is differentially sensitive to doxycycline, an inhibitor of mitochondrial protein synthesis (21), it is insensitive to the effects of low doses of cycloheximide, an inhibitor of translational elongation on cytosolic ribosomes. In most physiological contexts, this would be an unremarkable finding, as initiation, not elongation, is generally considered to be the rate-limiting step in translation. However, this is not the case in *Drosophila* larvae, where low doses of cycloheximide do restrain the growth of (*wild-type*) flies. This observation suggests that in the rapid growth conditions of the *Drosophila* larva, translational initiation has been maximally stimulated so that elongation has, indeed, become rate-limiting. This condition has been switched around in *tko^25t^*, where we postulate that translational initiation is already restrained by signalling through mTOR and other regulators. A different way of interpreting these data is to postulate a need for cytosolic and mitochondrial protein synthesis to remain in balance, to minimize the accumulation of excess unassembled or unfolded proteins that are surplus to requirements for mitochondrial biogenesis. Here, yeast provides a potentially informative model: mitochondrial and cytosolic protein synthesis and mitochondrial protein import are physiologically coordinated, minimizing proteotoxic stress in the mitochondrial matrix and potential overload of the chaperones and proteases that function to maintain protein homeostasis (44–46). In an extreme situation, failure of these homeostatic mechanisms could have a variety of drastic consequences, including saturation of the mitochondrial protein import machinery, the accumulation of unincorporated polypeptides in the mitochondrial matrix or proteotoxic stress in other cellular compartments such as the endoplasmic reticulum (ER). 

Conversely, interference with mitonuclear protein homeostasis in *Caenorhabditis elegans*, via knockdown of mitoribosomal proteins or treatment with inhibitors of mitochondrial translation, has been reported to extend lifespan. This has been attributed to a hormetic mechanism involving the activation of the mitochondrial unfolded protein stress response (47). Although the observation appears to contradict those in *Drosophila*, both results can be interpreted to indicate that maintaining a proper balance between cytosolic and mitochondrial protein synthesis is important for the organism to thrive. This is further illustrated by the finding that developmental time to eclosion and other life-history traits vary according to different combinations of mitochondrial and nuclear genotype in *Drosophila*, and this is further subject to nodulation by diet (48–51). The crucial regulator of cytosolic protein synthesis, 4EBP has also been reported to modulate survival and lifespan in *Drosophila* in response to cold temperatures, in ways that correlate with the effects on mitochondrial protein synthesis (52), likely reflecting the same underlying homeostatic process.

## Wnt Signalling of Mitochondrial Dysfunction

Studies in the model nematode, *C. elegans*, have revealed the existence of a systemic signal emanating from neurons, which induces the mitochondrial unfolded protein response UPR^mt^ in distant tissues (53). The primary signal is produced as a result of mitochondrial dysfunction in the affected tissue, and is believed to serve a protective function by alerting other tissues in the worm to the metabolic threat and preconditioning their response to it, much as the hypothesized metabolic or endocrine signal in *tko^25t^*. In the natural environment, the threat is likely to be toxicological in nature, *e.g*. the presence of cyanide or other RC inhibitors. As the nematodes are phyletically a sister group to the arthropods (54), the two processes might be predicted to share a common physiological mechanism. The nematode UPR^mt^-propagating signal has been tentatively identified as a ligand of the Wnt family (EGL-20), which is normally retained within the Golgi by the action of the retromer machinery. The endosomal retromer complex recycles the Wnt secretion factor MIG-14 from the plasma membrane back to the Golgi (55). Although it is not excluded that other members of the Wnt family could act similarly or in an accessory role, EGL-20 is both necessary and sufficient for the non-cell-autonomous UPR^mt^. There are at least seven members of the Wnt family in the *Drosophila* genome, expressed, usually at low levels, in a great variety of tissue patterns, and implicated in a myriad of developmental processes, notably segment polarity, axon growth and the morphogenesis of gonads and diverse adult appendages. None of them is an orthologue of EGL-20. EGL-20 in *C.elegans* might therefore be a CNS-specific inducer of the mitochondrial stress response, rather than the extracellular factor that directly transmits it. If this is correct, we are left with the possibility that many different such inducers, with complex and varying tissue-specificities, could elicit a common response.

## The Role of PGC-1

The PGC-1 family of transcriptional co-activators has been widely invoked as ‘master regulators’ of mitochondrial biogenesis or of metabolic signalling connected with growth. In *Drosophila*, there is a single member of the gene family, *spargel* (*srl*), which simplifies its analysis, taking away considerations of functional redundancy or diversification. Published data support the proposed role of Spargel, which is required for normal growth, female fertility and insulin responsiveness. However, in our own work we found that over-expression of *spargel* has no impact on the mutant phenotype of *tko^25t^* (56) nor on that of *sesB^1^* (57). Whilst this suggests that (negative) growth signalling operating in these mutants may over-ride that contributed by Spargel, we also saw no increases in mRNAs for mitochondrial or other metabolic enzymes in samples from control flies run alongside *tko^25t^* (56). Moreover, germline knockdown of *spargel* produced embryonic lethality with a dramatic failure of cellularization and gastrulation, but no specific effects on mtDNA, whilst transcripts for mitochondrial OXPHOS subunits, which are normally down-regulated after the mid-blastula transition when new transcription starts to replace the maternal mRNA set, remained close to their maternal level. These findings indicate a more general role for maternally supplied Spargel in development, *e.g*. in the activation of zygotic transcription. PGC-1 family members in mammals are already known to partner with a number of different transcription factors, including the oestrogen receptor ERRα, the thyroid receptor, Interferon Regulatory Factor-4, PPARγ for which PGC-1 was originally named, and the growth/mitochondrial biogenesis-associated nuclear-respiratory factors 1 (NRF-1) and 2 (NRF-2 or GABP). Assuming the same applies to Spargel, it may be more appropriate to regard it as a booster of transcription in a wide variety of contexts, and that a specific connection to mitochondria, metabolism or growth regulation is a misappropriation.

## Pathways of Steroid Biosynthesis and Activity

The major life cycle stages in *Drosophila*, including the larval moults and the onset of pupariation, are regulated by cyclical changes in the level of the steroid hormone 20-hydroxyecdysone (20E). Insects do not possess enzymes for the *de novo* biosynthesis of sterols, so require a supply of these essential precursors for steroid production from dietary sources. Although not all details of the biosynthetic pathway of 20E are yet known, several of the key enzymes have been identified, and are located within the ER or the mitochondria and thus require sequential transfer of intermediates between these compartments for the full pathway to operate (58, 59). 20E is synthesized as an inactive precursor, ecdysone, in the prothoracic gland, from which it is released to be taken up by the tissues and processed to the active hormone by the mitochondrial cytochrome P450 Shade (ecdysone 20-hydroxylase). Shade and the other P450 enzymes involved in the ecdysone synthesis pathway are collectively known as the Halloween class of enzymes, because null mutants of any of them fail to synthesize the exoskeleton, and thus have a ‘ghostly’ appearance. They use NADPH as an electron donor which provides an intriguing potential link to our observations on NADPH depletion in *tko^25t^*, in particular when grown on high-sugar medium (6). One simple idea would be that NADPH availability limits the rate of synthesis of ecdysone in the prothoracic gland and the rate of its conversion to 20E in the tissues. This would fit the idea that mitochondrial insufficiency generates a systemic metabolic signal that impacts growth via an effect on NADPH in target tissues.

One other important enzyme of steroid metabolism is Scully, the *Drosophila* orthologue of mammalian HSD10, a member of the 17-beta hydroxysteroid dehydrogenase family (60, 61). In addition to its activity as a metabolic enzyme, Scully has a ‘moonlighting’ role in a completely different process, namely mitochondrial RNA metabolism. This second function, conserved up to mammals, requires the co-operation of two other proteins, the endonuclease Mulder and the methyltransferase Roswell, forming a heterotrimeric complex that acts as the mitochondrial RNase P (61), the processing enzyme responsible for site-specific cleavage at the 5′ end of tRNA precursors. Mulder and Roswell have no known functions in steroid metabolism, whilst the exact metabolic role of Scully remains to be documented in detail. In principle, it represents a potential link between mitochondrial gene expression and steroid production, processing and signalling.

Steroid hormones in *Drosophila* act as key regulators of growth and body size (62), with a ‘critical weight’ threshold (CW) reached during early third-instar (L3) stage (63). Nutrient deprivation prior to CW being attained leads to a decreased rate of growth mediated by IIS, but no change in the final body size. Conversely, nutrient deprivation after CW leads to decreased body size (64). Although ecdysone signalling takes over the primary role in growth regulation once CW is traversed, for a short period it co-operates with IIS, by virtue of activating the expression of DILP6 in the fat body, during the post-feeding period leading up to pupariation (64–66). This is believed to recalibrate body size according to the amount and nature of stored nutrients accumulated during the final period of feeding, thus integrating nutritional and steroid signals, in part by modulating the size of the prothoracic gland. A delay in the release of ecdysone during L3 means that larvae will exceed the normal CW before ceasing to feed, allowing them to attain a higher final size, whilst premature release of ecdysone from the prothoracic gland has the converse effect (67). The conversion of 20E to ecdysone by the mitochondrial cytochrome P450 Shade is dependent on nutritional signalling through the mTORC1 pathway (66), and a further layer of control is exercised via the release of DILP8 from imaginal discs that have suffered growth retardation, which delays ecdysone release and the onset of pupariation (68, 69). Steroid synthesis prior to the larval–pupal transition depends on the accumulation of dietary cholesterol into lipid droplets (LDs), whose formation requires the *Drosophila* somatic mitofusin Marf (70). Marf is needed for the fusion of mitochondria, as well as their interactions with the ER and LDs.

The developmental effects of ecdysone are mediated by a heterodimeric complex comprising the ecdysone receptor (EcR) and ultraspiracle. EcR appears to be preset in multiple cellular compartments, and to shuttle dynamically between them, ad has been proposed to exert non-genomic effects outside of the nucleus (71, 72), including a reported presence in mitochondria in one other species of insect (73), based on immunocytochemistry and subcellular fractionation. The presence in mitochondria of isoforms of many nuclear transcription factors, including nuclear receptors, has been reported for over 20 years, *e.g*. (74), but is still regarded with some scepticism. Such factors may participate in transcription (74) or in other, as yet undetermined processes. In the case of steroid hormones such as ecdysone, which are partly synthesized in mitochondria, such observations suggest a novel type of cross-talk that remains to be explored. Circumstantial evidence for a mitochondrial role of ecdysone signalling has come from a recent finding that functional deficiency of the mitochondrially localized Aarf domain-containing kinase 1 produces a phenotype that resembles the effects of attenuated ecdysone supply (75).

## Concluding Remarks

Growth is a fundamental aspect of animal development, for which the *Drosophila* larva presents an idealized model. Mitochondria play crucial roles in enabling larval growth, by converting substrates into new biomass. However, their capacity for doing so is not unlimited, and is potentially influenced by nutrient availability, by toxins, xenobiotics or antibiotics, by hypoxia, or by nuclear-mitochondrial incompatibilities. Metabolic co-operation between cells is vital for mitigating these burdens but, in addition, is likely to involve signalling systems that attune protein synthesis in different cells and subcellular compartments. In turn these should minimize proteotoxic stress, maintain redox balance, ensure adequate bioenergy supply and integrate growth processes in different tissues and organ systems. As is clear from this review, we are still only just beginning to elucidate the nature of these processes and of the molecular machinery that executes them.

## Abbreviations

CW: ‘critical weight’ threshold
EcR: ecdysone receptor
IIS: insulin/insulin-like growth factor signalling
LD: lipid droplet
mtDNA: mitochondrial DNA
OXPHOS: oxidative phosphorylation
RC: respiratory chain.
